# Development, validation and psychometric evaluation of the Chinese version of the biopsychosocial impact scale in orofacial pain patients

**DOI:** 10.3389/fpsyg.2023.1101383

**Published:** 2023-03-07

**Authors:** Ze-Yue Ou-Yang, Yao Feng, Dong-Dong Xie, Yi-Fan Yang, Yun Chen, Ning-Xin Chen, Xiao-Lin Su, Bi-Fen Kuang, Jie Zhao, Ya-Qiong Zhao, Yun-Zhi Feng, Yue Guo

**Affiliations:** ^1^Department of Stomatology, The Second Xiangya Hospital of Central South University, Changsha, Hunan, China; ^2^The Medical Psychological Institute, The Second Xiangya Hospital of Central South University, Changsha, Hunan, China

**Keywords:** chronic orofacial pain, scale development, Chinese patients, factor analysis, measurement invariance, clinical responsiveness

## Abstract

**Background:**

The objective of this study was to develop the Chinese version of the biopsychosocial impact scale (BPIm-S) to assess functional limitation and psychosocial distress in orofacial pain (OFP) patients in mainland China, and investigate the factor structure, reliability and validity, measurement invariance, as well as scores differences across genders, age and educational status among OFP patients.

**Methods:**

The BPIm-S was developed and evaluated in four stages: (1) concept selection and item generation; (2) a pilot study assessing face and content validity; (3) the factors structure, reliability, convergent validity, and measurement invariance; and (4) concurrent validity and clinical responsiveness. Exploratory (EFA) and confirmatory factor analyses (CFA) were performed on data gathered from 406 OFP patients to assess construct validity. Composite Reliability (CR) and the Average Variance Extracted (AVE) were used to assess internal convergent validity. CR, internal consistency, and split-half reliability were also performed to determine the reliability. Multigroup CFA (MGCFA) was used to assess measurement invariance across genders, age and educational status. Mann–Whitney test compared scores across different genders, age and educational status. Participants completed the BPIm-S, visual analog scale (VAS), brief pain inventory facial (BPI-F), General Anxiety Disorder-7 (GAD-7) and Patient Health Questionnaire-9 (PHQ-9), and spearman’s correlation coefficient was used to evaluate the concurrent validity and item-total correlations. A total of 12 patients with OFP completed the BPIm-S twice to test clinical responsiveness. To conduct the CFA and measurement invariance analysis, Mplus 8.4 was used. IBM SPSS Statistics 21 software and SPSSAU, a web-based data science algorithm platform tool, were used for all additional studies.

**Results:**

For the preliminary version, 17 items were chosen. A total of four items were removed following the pilot research. The remaining 13 items of the BPIm-S comprised an overall summary scale. Excellent reliability (Item-to-total correlations ranged from 0.763 to 0.912) and strong internal consistency (Cronbach’s α = 0.970, functional limitation, 0.962, and psychosocial distress, 0.977) were discovered. CFA also validated the structural validity of the 13-item scale. EFA was performed and a two-factor structure was investigated. In addition, MGCFA corroborated the measurement invariance of the BPIm-S across gender, age, and educational status. Patients over the age of 30, those with a medium level of education, and those with a low level of education showed substantially greater levels of functional limitation and psychological distress (Wilcoxon test, *p* < 0.001). Both concurrent validity and clinical responsiveness were assessed to be of good quality.

**Conclusion:**

The BPIm-S demonstrated good psychometric qualities and is a reliable tool that can now be used by clinicians to evaluate functional limitation and psychosocial distress among OFP patient.

## 1. Introduction

Oral health is a critical component of general health (Petersen and Kwan, 2004). Oral health-related quality of life (OHRQoL) refers to the role of oral conditions or diseases on quality of life, which is closely related to the impact of pain or discomfort, physical, psychological, and social functions on wellbeing (Geels et al., 2008). Various studies have shown a connection between periodontal disease and other, more serious health issues, including endocarditis, stroke, and diabetes (Hiraki et al., 2020). Depression, anxiety is only some of the psychiatric issues that can coexist with burning mouth syndrome (Stohler, 2001; Kim et al., 2020). Throughout the world, increasing attention has been paid to the relationship between oral health and general health, including physical and mental health, particularly in China as research and policy regarding oral health have developed (Petersen, 2003). It has been reported that the Chinese government has released a series of policies on health, including the Healthy China 2030 blueprint and the Chronic Diseases Program in 2017 (Kong, 2017; Li and Chen, 2020; Li et al., 2020). These policies all involve oral health promotion as one of their main components. They also aim to encourage the development of oral health behaviors and increase the public’s oral health literacy.

Orofacial pain (OFP) is one of the most complained about oral and maxillofacial problems. The global prevalence of OFP is estimated to be between 14% and 42% (McMillan et al., 2010). And it is associated with healthcare costs, loss of productivity and reduced quality of life, with a high social and personal burden (Durham et al., 2016). Although the epidemiology of OFP have been well-studied in many countries, limited surveillance data are available in China (Leung et al., 2008). This suggests that OFP would be the area of great concern and profound impact on oral health and general health in China. Moreover, OFP is a typical type of psychosomatic disorder in oral diseases (Shamim, 2014). Long-term OFP has been demonstrated in studies to be associated with sleeping issues, cardiovascular problems, indicating that OFP may be part of a general health condition (Lee and Auh, 2022). Also, approximately 30% of OFP patients exhibit psychiatric symptoms, which often go undetected and untreated (Toyofuku, 2016). Thus, it is important to identify patients with OFP at an early stage and to treat them with appropriate diagnosis.

The International Headache Society (IHS) published the International Classification of Orofacial Pain, the first edition (ICOP) in 2020 (IOP, 2020). ICOP proposed that in addition to traditional biological factors, psychosocial factors are not only powerful predictors of pain, function and quality of life of patients with chronic pain. Additionally, psychological variables are strong predictors of reactions to medical treatments such as pain relief surgery and medication. In the course of the illness of OFP patients, complex psychological conditions often aggravate the distress of the disease (Festa et al., 2021), but the existing research on this model is not deep enough, which has brought great obstacles to clinical diagnosis, intervention and treatment. Therefore, the biopsychosocial model has become the most comprehensive model in the field of OFP management.

Given that biopsychosocial disabilities have been observed in patients with OFP (Randall et al., 2016). The measurement of biopsychosocial disturbance is also an integral part of OFP assessment. It is critical for clinicians to take into account patient-reported outcome measures (PROMS) when diagnosing and evaluating treatment outcomes (Kyte et al., 2015). Current PROMS that measure function and disability in patients with OFP including Mandatory Function Improvement Questionnaire (MFIQ) (Stegenga et al., 1993), Craniofacial pain and disability inventory (CF-PDI) (Madrid et al., 2014), The 8-item and 20-item Jaw Functional Limit Scale (JFLS) (Ohrbach et al., 2008) and so on, which most focus on physical pain and disabilities but lack of the psychosocial dimension. Compared with the above instruments, although the Manchester Orofacial Pain Disability Scale (MOPDS) graded the degree of both physical and psychosocial disabilities, it still is lack of characteristic tests, like measurement invariance, clinical responsiveness and so on (Aggarwal et al., 2005; Kallás et al., 2013). The Consensus-Based Standards for the Selection of Health Measurement Instruments (COSMIN) proposed that PROMS should have validity, reliability and responsiveness (Prinsen et al., 2018). Due to the large number of OFP people on the Chinese mainland, research into the use of PROMS in this population is essential. This questionnaire should include the biopsychosocial dimension to reflect the functional limitation and psychosocial distress of OFP patients. Pain assessment involves the use of subjective and objective measures and the subjective measures involve the use of diagnostic daily pain diary where patients verbalize or describe their pain. Objective measures include clinicians observing the patient’s response to pain according to PROMS, such as the extent to which aspects of life are affected by pain or psychological changes (IOP, 2020). PROMS assessment must be performed systematically and using rigorously validated questionnaires to minimize the non-physiological variability inherent in such measures. Therefore, a psychometric validated PROMS that can be used to assess symptoms, related functions and the impact of OFP on quality of life in OFP patients is very necessary.

### 1.1. Assessing the OFP

The comprehensive evaluation of physical and psychological variables needs to rely on accurate screening, and use a relatively short, accurate instrument that can be used by people with different characteristics but studies using unverified scales are prone to the risk of bias (Marshall et al., 2000). In addition, among OFP patients, their perception of OFP is vulnerable to various social and cultural factors (Lin et al., 2013), so it is recommended to make cultural modifications to prevent cultural bias. There is a need for a Chinese scale able to assess the physiological and psychosocial dimensions of Chinese OFP patients, and its structure is explored and verified to make the scale more useful.

Many studies have shown that OFP is related to gender, age and different educational status. Shinal and Fillingim (2007) pointed out that women of childbearing age are more likely to have OFP than men. Dussor et al. (2018) proposed that the oral and facial morbidity of men and the elderly is high. In addition, our previous research has also proved that education will also affect OFP, for example, the high incidence of OFP can be observed among college students (Feng et al., 2022). Given the above results, it cannot be ruled out whether the population is affected by latent variables (gender, age, education, etc.) because they didn’t use the same and accurate assessment instruments. Therefore, the scale should provide measurement invariance data (Kline, 2015). Appropriate assessment instruments contain measurement invariance, which indicates that personal traits unrelated to the structure evaluated by the scale do not influence individual project ratings (Gregorich, 2006). Psychological test score differences are meaningful after assessing the scale’s measurement invariance in gender, age, and education. We aim to explore a suitable model structure, and test the reliability of the scale, especially after in-depth analysis of its invariance in terms of gender, age and educational status, to compare the differences between groups, which could make sure that the scale could be utilized as an important tool for assessing the impact of OFP and help doctors to make individualized clinical treatment.

PROMS represents an important measure of the impact of illness and its treatment on symptoms and functions. The questionnaire score should respond to the clinically obvious disability, that is, certain changes in the questionnaire score should reflect the corresponding changes in the clinical situation (Gillespie et al., 2014). Therefore, it is necessary to test the clinical reactivity of the newly developed PROMS.

In this study, we built on past research to create and test a new scale to measure the functional restriction and psychological effect of OFP on patients’ lives. This research aimed to create a simple assessment scale for OFP patients that could be used to evaluate the complex impact of OFP on patients’ everyday life and assist physicians in ordinary clinical practice.

## 2. Materials and methods

### 2.1. Theoretical framework

The ICOP guidelines suggest that the biopsychosocial model is strongly embedded as a concept in the understanding and assessment of OFP (IOP, 2020). The model suggests that OFP is increasingly understood as a complex biopsychosocial phenomenon that is highly associated with physical disability as well as a high prevalence of psychosocial distress. Physical disability (functional limitation) is reflected in the impact on quality of life related to oral health, i.e., chewing, mouth opening and speaking, in addition to the impact on life activities (Liu et al., 2021). And psychosocial distress demonstrated that patients with OFP often suffer from anxiety and depression (Wang et al., 2015).

### 2.2. Participants

This research was authorized by the Experimentation and Ethics Committee of the Second Xiangya Hospital of Central South University (KQ2019FY01). The study was conducted in compliance with the tenets of the Declaration of Helsinki. Participants in this research were recruited from the Department of Stomatology at Central South University’s Second Xiangya Hospital, and all gave their permission before to participation. People who are fluent in Chinese are eligible. According to ICOP criteria (IOP, 2020), participants had to be diagnosed with definite OFP. The diagnostic criteria were validated by OFP physicians, temporomandibular doctors and endodontics experts according to the ICOP criteria (Table 1). The exclusion criteria for clinical samples were: (1) those who could not read and understand the scale correctly; (2) oral cancer patients; and (3) any other concurrent Axis I disorders according to the Diagnostic and Statistical Manual of Mental Disorders, fifth edition (DSM-5) (American Psychiatric Association, 2013); any organic brain disorder, severe head trauma, or history of substance abuse.

**TABLE 1:** Diagnostic criteria for OFP used in the studies^a^.

Criteria^b^
1. OFP for at least 3 months (considered as chronic pain, according to the ICOP)
2. Baseline pain score ≥3 on a ten-point visual analog scale (VAS)

^a^The criteria complied with those defined by the ICOP.

^b^OFP severity is considered abnormal when either (1) or (2) applied.

### 2.3. Measurement

#### 2.3.1. Instrument development

**FIGURE 1:**
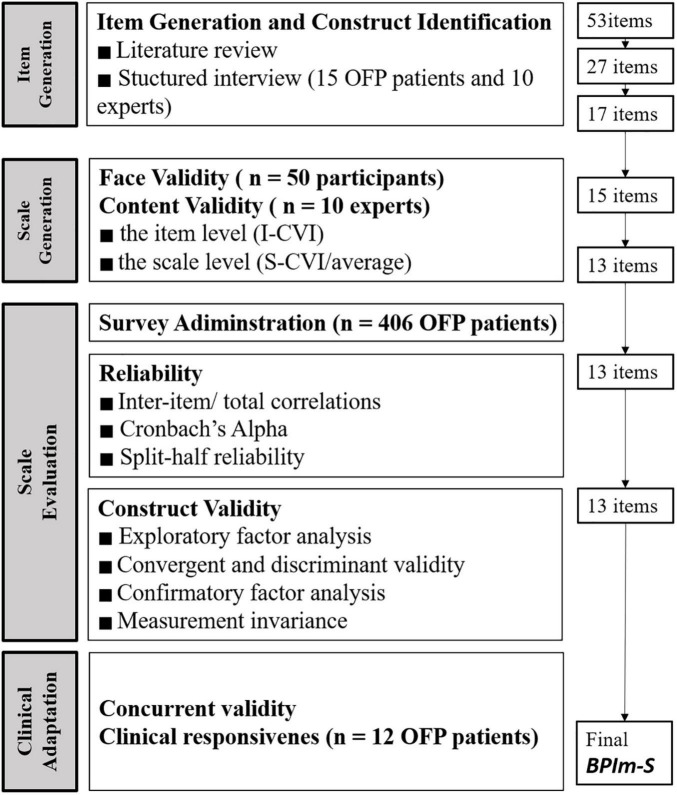
Phases in the development of the scale.

According to Boateng et al. (2018) development and psychometric testing of scale is one of most critical in much of the work of health, social, and psychological sciences. It includes four stages: (1) concept is selected and items are generated; (2) the scale is constructed; (3) the factors of the scale are captured, reliability, validity, and measurement invariance are tested, and compare the scores of different sociodemographic characteristics; and (4) clinical adaptation is assessed. The biopsychosocial impact scale (BPIm-S) was developed using the exploratory sequential research design to assessed the OFP health life related functional-psychosocial quality (Figure 1).

#### 2.3.2. Stage I concept selection and item generation

##### 2.3.2.1. Reviews of previous qualitative insights and OFP literature

On the basis of a survey of the relevant literature, current theories and models, and accessible measuring techniques, a precise conceptualization of the notion was first formulated (Boateng et al., 2018). We had a literature review related to OFP, trigeminal neuralgia, epidemiology, maxillofacial pain, temporomandibular, primary headache and so on, which were searched for in PubMed, China National Knowledge Infrastructure (CNKI) and other databases (Supplementary Table 1). Items based on the articles’ functional and psychological views on OFP were developed and extracted. The item pool of 53 items was generated from the literature review and personal interviews.

##### 2.3.2.2. Structured interview by target population and experts

Individuals interview were then performed with patients having a verified diagnosis of OFP in order to find observable manifestations of the idea, as opposed to depending only on a theoretical perspective. The participants (*n* = 15 patients with OFP) were later interviewed, and the following topics were covered: overall impression, thoroughness of instructions, and understandability of the questionnaire. They were also questioned whether it addressed all significant components of their pain-related life discomfort. Two hypothetical conceptual dimensions, functional distress and psychological distress, were derived from the examination, comparison, and combination of the original 27 items.

Further screening of items experts in dentistry, rehabilitation medicine, epidemiology, biostatistics, sociology, and psychology were invited to further screen the items. A total of 17 items were finally retained, which served as the first draft of the questionnaire (BPIm-S first draft).

#### 2.3.3. Stage II scale development

##### 2.3.3.1. Face validity–Evaluation by target population

To assess whether the questions reflected the study domain and met the necessary criteria, to confirm that the questions in the generated scale were appropriate and understandable to the targeted respondents, a cognitive interview was performed with 50 OFP patients before the survey was sent.

First-round BPIm-S completion times averaged 3 min and 19 seconds across participants. Items that were not part of the OFP, items that overlapped with other items, and items with confusing representations were removed. Using the results of the cognitive interviews, we revised the grammar and the available responses. More than seventy percent of patients replied “not relevant” to two questions about behavior disturbance (not doing chores and eating more often).

##### 2.3.3.2. Content validity–Evaluation by experts

Delphi methodology was used to conduct experts investigation in this study. Ten specialists examined the scale to see whether the generated items adequately measured the targeted variables. The research group included two OFP physicians (Guo yue, He Zhi-jing), two temporomandibular doctors (Feng Yun-zhi, Liu Yin-chen), one anesthesia specialist (Wang Ya-ping), two endodontics experts (Gao Yi-jun, Li Wen-hui), two psychosocial research scientists (Chen Jin-dong, Yuan Hui), a statistician (Zhou Ying-hui). Through the whole process of refining and concluding the questionnaire, they contributed valuable insight and input.

The content validity index (CVI) was calculated at both the item (I-CVI) and scale (S-CVI/average) levels as part of the evaluation of the scale’s content validity. Ten experts used a four-point scale ranging from 1 (not relevant) to 4 (very clear) to assess the relevance and clarity of the underlying topic or concept. Item relevance was determined using the Polit and Beck-proposed value range, whereby an I-CVI > 0.78 indicates relevance and an S-CVI of 0.80 or more indicates an appropriate scale (Polit and Beck, 2006).

Two items (items 9 and 11) were eliminated for the value range recommended by Polit and Beck (2006) to determine an item’s significance. Some items were also changed to improve their clarity based on the opinions and recommendations of the experts. Item 13 “Have you ever been recommended for help because of OFP” had little to do with psychology. Item 17 “Have you ever felt punished for OFP (sense of punishment)” seemed to be difficult for Chinese people to understand, as the topic is more likely to reflect the psychosocial distress of theistic believers. Thus, items 9 and 11 had a confusing representation were removed (Supplementary Table 2). The S-CVI/AVE of the 13-item of BPIm-S was 0.954 points.

#### 2.3.4. Stage III scale evaluation

##### 2.3.4.1. Participant recruitment

The 406 participants met the requirements of being at least 18 years old and Chinese-literate. Any patients who met the ICOP criteria for OFP were included in the study. The doctor will decide if the OFP patient needs additional assessment or referral to a specialist care facility.

##### 2.3.4.2. Reliability

###### 2.3.4.2.1. Item analysis and item-total correlations

We calculated the average, standard deviation, minimum, and maximum for each item. The data normality was examined using the Shapiro-Wilk test (Kim, 2012).

We used the theory of classical testing (CTT) to estimate inter-item and total item correlations, which is used to check the relationships that exist between the items in the pool (Crocker and Algina, 1986).

###### 2.3.4.2.2. Exploratory factor analysis

Exploratory factor analysis (EFA) was used to test the underlying structures within the BPIm-S. For EFA, 5 or 10 subjects per item are recommended regardless of the number of items (Gorsuch, 1990). To ensure that EFA and confirmatory factor analyses (CFA) were performed independently (Lee, 2016), 193 subjects were selected using IBM SPSS Statistics’ random sampling method for EFA (Child, 1990).

###### 2.3.4.2.3. Convergent validity

Both the convergent validity of the measure were assessed using the method developed by Fornell and Larcker (1981). If there is a high average variance extracted (AVE) and composite reliability (CR) between the scale’s items, then the convergent validity of the scale is established.

###### 2.3.4.2.4. Confirmatory factor analysis

We tested the factorial structure obtained by the EFA with the remaining 213 were used for CFA sample using CFA.

###### 2.3.4.2.5. Factorial invariance across genders, age, and education level

Multigroup CFA (MGCFA) was used to probe the feature of measurement invariance (Munro, 2005). CFA’s model was used in these measurement invariance tests. MGCFA allows users to evaluate the relative merits of various degrees of model constraint. Age, gender, and status of education were the demographics studied, and four tiers of measurement invariance were examined. The following degrees of invariance were examined as part of the analyses: First, the concept of “configural invariance,” which indicates that there is no significant difference in the clustering of items and the factors that they represent across groups; second, “metric invariance,” which indicates that factor loadings are comparable across groups; third, “scalar invariance,” which indicates that intercept are comparable across groups; and fourth, “residual invariance,” which indicates that the residual variances are not significantly different across groups.

#### 2.3.5. Stage IV clinical adaptation

##### 2.3.5.1. Concurrent validity–Evaluation through scales

Concurrent validity refers to a measure’s capacity to identify a simultaneously evaluated criteria (Bowen and Masa, 2015). We examined the concurrent validity of BPIm-S questionnaire against other commonly used scales for assessing functional limitation and psychosocial distress. A total of five questionnaires were filled out by the participants: the final 13-item version of the BPIm-S, brief pain inventory facial (BPI-F), MOPDS, General Anxiety Disorder-7 (GAD-7), and Patient Health Questionnaire-9 (PHQ-9). The BPI-F is a measure of facial functions (Sandhu et al., 2015), the MOPDS was found to be reliable to evaluate the functional limitation (Aggarwal et al., 2005), while the GAD-7 (Spitzer et al., 2006), and PHQ-9 (Smarr and Keefer, 2011) were well-validated tools used to screen and diagnose generalized psychosocial disorder in clinical practice. It was assessed by evaluating the spearman correlation coefficients between the BPIm-S score and the scores of the BPI-F, MOPDS, GAD-7, and PHQ-9.

##### 2.3.5.2. Clinical responsiveness–Evaluation by patients

A total of 12 patients with OFP (3 females, 9 males; median age, 25 years) (P25, P75: 22, 52; type of OFP disease: migraine, toothache, tension and maxillofacial headache, burning mouth syndrome) who underwent physical therapy (hot compress) were collected for evaluating the clinical responsiveness. Hot compress treatment was performed in six sessions of 30 min duration each before bedtime, three times per week for 2 weeks. All participants were able to answer questionnaires without assistance.

### 2.4. Data analysis

In the structured interview by target population and experts’ phase, OFP patients were interviewed by the dentists and their responses were collated. The experts scored each item based on its relevance to the OFP, its objective measurability, and its scientific interpretation as well. The numbers 3, 2, and 1 indicate “consistent,” “general,” and “inconsistent,” respectively. Items with a mean <2.000 and a coefficient of variation (CV) greater than 0.400 are excluded from the analysis. Then we conducted face and content validity tests on the first draft of the BPIm-S. A CVI calculation will be used to determine the face validity of the assessment (Petrick, 2002). Finally, a 13-item of BPIm-S was formed.

In the item analysis and item-total correlations phase, to determine if the data distribution is normal, the Shapiro-Wilk test was used. Data with a *p*-value of greater than 0.05 are considered to fit a normal distribution (α = 0.05) (Kim, 2012). There were four different types of analysis performed on each item: the median, (P25, P75), minimum, and maximum. Internal reliability index was calculated using Cronbach’s α. Internal consistency reliability assesses the homogeneity of items belonging to the same scale or domain, which was estimated using Cronbach’s alpha (α ≥ 0.7, acceptable), and split-half reliability (*r* ≥ 0.7, acceptable) (Cohodes et al., 2022).

Following item selection and reliability analyses, an EFA was run in IBM SPSS Statistics 21 software and CFA were run in Mplus 8.4. version. Kaiser-Meyer-Olkin and Bartlett tests were used to determine the adequacy of the sampling of the EFA (Vetterlein et al., 2022). Then, the latent factors of the BPIm-S were extracted *via* the maximum-likelihood EFA with varimax rotation. The number of extractable factors was determined using parallel analysis. The 3-indicator rule stipulates that each factor must have at least three items. We removed items with communality values less than 0.2.

Next, discriminant validity was assessed with the web-based data science algorithm platform tool SPSSAU. The AVE > 0.7, CR > 0.5 indicating good convergent validity, and the square root of AVE is greater than the correlation coefficient between the factors, indicating a good discriminant validity of the test.

Confirmatory factor analyses and measurement invariance analyses were performed with Mplus 8.4 version the χ2 statistic, standardized root mean square residual (SRMR), a Tucker-Lewis index (TLI), and a root mode square error of approximation (RMSEA) were used to estimate the model fit. Following Bryne (Byrne, 2011), we considered the fit of the factorial model to the data was considered adequate when CFI and TLI ≥ 0.90. In addition, SRMR < 0.05 and RMSEA ≤ 0.1 were considered to indicate a satisfactory fit (Steiger, 1990). In MGCFA, Goodness-of-fit statistics were estimated for each model and for each model relative to the previous, less restricted, model. The fit of the model was assessed using the CFI, TLI, SRMR, and RMSEA fit indices. we evaluated ΔCFI and ΔRMSEA between the more and less constrained models. ΔCFI and △TLI larger than 0.01 and ΔRMSEA larger than 0.015 indicated a significant worsening of fit (Chen, 2007).

Concurrent validity was assessed by evaluating the spearman correlation coefficients between the BPIm-S score and the scores of the BPI-F, MOPDS, GAD-7, and PHQ-9. A total of 12 patients with OFP who underwent physical therapy (hot compress) were collected for evaluating the clinical responsiveness. All participants were able to answer questionnaires without assistance. The scores before and after the hot press treatment were compared by using the 2-tailed paired Mann–Whitney test to evaluate responsiveness. The significance level was set at 0.05.

## 3. Results

### 3.1. Demographic characteristics

**FIGURE 2:**
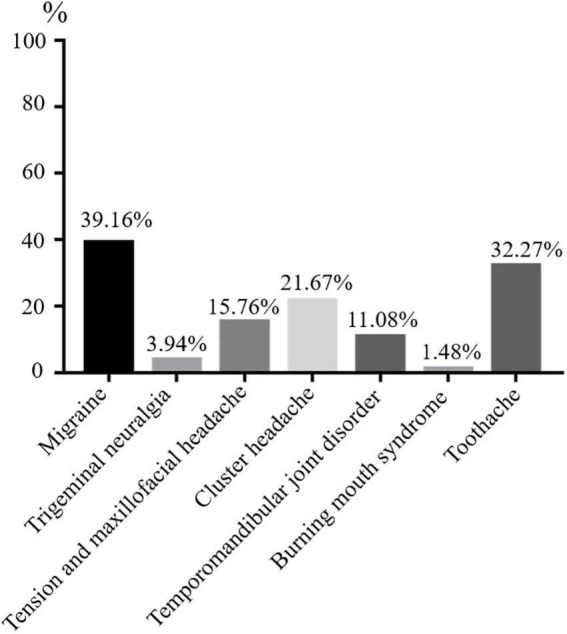
Diagram of OFP patient distribution.

A total of 406 participants were recruited for scale evaluation in stage III of this study [Median age (P25, P75), 34 years (24, 52)], The doctor will decide if the OFP patient needs additional assessment or referral to a specialist care facility. More than 50% of respondents were female (*n* = 241) and over 30 years old (*n* = 220). Regarding the education level of the participants, 214 participants (52.71%) were high education and 192 (47.29%) graduated from high school or lower (Table 2 and Supplementary Table 3). The most frequently diagnosis of OFP was migraine (*n* = 159), followed by toothache (*n* = 131), cluster headache (*n* = 88), tension and maxillofacial headache (*n* = 64), temporomandibular joint disorder (*n* = 45), trigeminal neuralgia (*n* = 16), and burning mouth syndrome (*n* = 6) (Figure 2).

**TABLE 2:** Characteristics of validation study participants (*N* = 406).

	Median-age, (interquartile range)	*N* (%)/(95% CI)
**Age (years)**		
	34 (24, 52)	
**Gender**		
Male		165 (40.6%)/(35.7%,45.3%)
Female		241 (59.4%)/(54.7%,64.3%)
**Education status**		
High education (undergraduate and above)		214 (52.7%)/(48.0%,57.6%)
Medium and low education (high school and junior high school)		192 (47.3%)/(42.4%,52.0%)

### 3.2. Item analysis and item-total correlations

In this population sample, the Shapiro-Wilk test of normality of distribution did not indicate a normal distribution (*W* = 0.897, *p* < 0.05). The BPIm-S comprises 13 items that describe 2 dimensions: functional limitation, psychosocial distress. The format for the questions is “Have you ever had difficulty concentrating because of orofacial pain” The items are graded on a five-point Likert scale, from 0 (never) to 4 (very frequently). The median BPIm-S score was 25 (13, 31) (range 13–52). Item-total correlations analysis was performed using the spearman correlation index. Adequate spearman correlation was found between the items and the whole, with values ranging from 0.763 to 0.912 (Table 3).

**TABLE 3:** Descriptive statistics of the responses given to the items of the BPIm-S by the participants.

Item	Median	Interquartile	Min	Max	Item-total correlations	Cronbach’s α if item deleted
Item 1	2	(1, 2)	1	4	0.799	0.968
Item 2	2	(1, 2)	1	4	0.776	0.969
Item 3	2	(1, 2)	1	4	0.774	0.969
Item 4	2	(1, 2)	1	4	0.763	0.970
Item 5	2	(1, 2)	1	4	0.796	0.969
Item 6	2	(1, 3)	1	4	0.892	0.967
Item 7	2	(1, 3)	1	4	0.904	0.967
Item 8	2	(1, 3)	1	4	0.906	0.966
Item 9	2	(1, 3)	1	4	0.883	0.967
Item 10	2	(1, 3)	1	4	0.875	0.967
Item 11	2	(1, 3)	1	4	0.912	0.966
Item 12	2	(1, 3)	1	4	0.879	0.967
Item 13	2	(1, 3)	1	4	0.889	0.967

### 3.3. Reliability analysis

After factor analysis, the overall Cronbach’s α value for the scale was 0.970, indicating a very high degree of reliability. Cronbach’s α would decrease if any of the 13 items were removed (Table 3). Cronbach’s α scores for the two variables were as follows: 0.962 for functional limitation and 0.977 for psychological suffering. The internal consistency of scale was determined by split-half reliability. The split-half reliability coefficient of FPIm-S was 0.880. They were deemed satisfactory for both the overall score and the dimension scores.

### 3.4. Exploratory factor analysis

**FIGURE 3:**
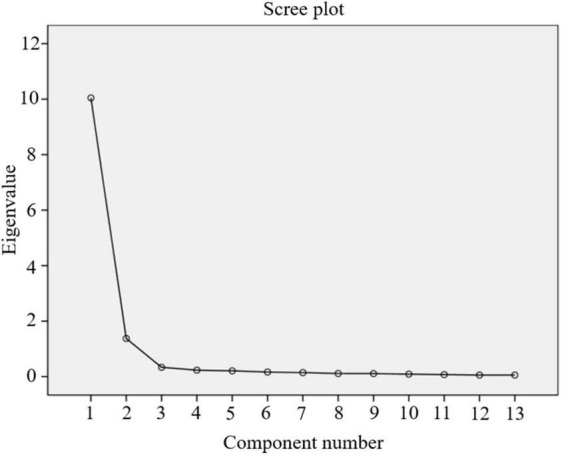
The scree plot of the BPIm-S factors.

The remaining 13 scale items’ underlying variables were uncovered by EFA. Kaiser-Meyer-Olkin (KMO) value of 0.945 (*p* < 0.001) was found in the preliminary factor analysis, which is above the minimum required value of 0.5 and shows the sufficiency of scale items for factor analysis. Bartlett’s test of sphericity also verified the factorability of the 13 items (χ^2^ = 3818.156; *p* < 0.01). Figure 3 indicates that 2 components were moderately distinguishable and explained 87.845% of the variance. As reflected in Table 4, factor 1 had 5 items, and factor 2 had 8 items for a total of 13 items. Also, the factor loading of each item were above 0.8 in their dimensions.

**TABLE 4:** Exploratory factor analysis of the BPIm-S scale items.

The BPIm-S domain	Item	Factor 1	Factor 2
**Functional limitation**			
	Item 1	0.825	
	Item 2	0.866	
	Item 3	0.859	
	Item 4	0.878	
	Item 5	0.806	
**Psychosocial distress**			
	Item 6		0.830
	Item 7		0.858
	Item 8		0.877
	Item 9		0.807
	Item 10		0.883
	Item 11		0.887
	Item 12		0.861
	Item 13		0.865

### 3.5. Construct validity evaluation

Construct validity was established *via* convergent validity. By engaging the AVE and CR, the convergent validity was tested. The CR was 0.927 for functional limitation and 0.957 for psychosocial distress. The AVE was 0.718 for functional limitation and 0.738 for psychosocial distress. AVE scores for all variables were greater than 0.50 and lower than the CR, hence establishing convergent validity.

### 3.6. Confirmatory factor analysis

The CFA findings indicated that a two factor model provided an excellent sufficient fit to the OFP topics, and the results were as follows: χ^2^ = 142.641, *df* = 64, χ^2^/*df* <5, CFI = 0.956, TLI = 0.947, SRMR = 0.036 and RMSEA = 0.076.

### 3.7. Measurement invariance across genders, age, and education level

Table 5 shows the fit measures of the multi-group models for testing measurement invariance across age, genders and education level. Considering genders, the data were extremely well matched by the two-factor model configural invariance model (CFI = 0.960, TLI = 0.951, SRMR = 0.033, RMSEA = 0.075). A satisfactory fit was shown using a limited metric invariance model (CFI = 0.957, TLI = 0.951, SRMR = 0.038, RMSEA = 0.074). The data were well-fitted by the scalar invariance model (CFI = 0.954, TLI = 0.952, SRMR = 0.038, RMSEA = 0.074). Last but not least, the residual invariance was compared to the scalar invariance, suggesting that invariance remained constant with each additional model constraint (CFI = 0.949, TLI = 0.951, SRMR = 0.037, RMSEA = 0.075), suggesting that measurement invariance may be considered to be across genders. Similar results were found for age and educational status indicating that the structure, factor loadings and item intercepts are invariant across age and educational status. The measurement invariance held when the fitting change met the following conditions: △CFI ≤ 0.01, △TLI ≤ 0.01 and △RMSEA < 0.015 (Table 5).

**TABLE 5:** Model comparisons for measurement invariance testing across gender, age, education level groups in OFP patients (*N* = 406).

Model	χ^2^ (*df*)	CFI	TLI	SRMR	RMSEA	ΔCFI	ΔTLI	ΔRMSEA
**Invariance across gender groups**								
Model 1	272.934 (128)	0.960	0.951	0.033	0.075			
Model 2	295.386 (139)	0.957	0.951	0.038	0.074	−0.003	0.000	-0.001
Model 3	316.008 (150)	0.954	0.952	0.038	0.074	−0.003	0.001	0.000
Model 4	347.823 (163)	0.949	0.951	0.037	0.075	−0.005	-0.001	0.001
**Invariance across age groups**								
Model 1	299.855 (128)	0.950	0.939	0.034	0.081			
Model 2	321.545 (139)	0.947	0.940	0.044	0.080	−0.003	0.001	-0.001
Model 3	341.483 (150)	0.944	0.942	0.045	0.079	−0.003	0.002	-0.001
Model 4	373.855 (163)	0.938	0.941	0.046	0.080	−0.006	-0.001	0.001
**Invariance across educational status groups**								
Model 1	271.658 (128)	0.956	0.946	0.035	0.074			
Model 2	292.479 (139)	0.953	0.947	0.046	0.074	−0.003	0.001	0.000
Model 3	314.279 (150)	0.950	0.948	0.049	0.073	−0.003	0.001	-0.001
Model 4	329.055 (163)	0.949	0.951	0.050	0.071	−0.001	-0.003	-0.002

Model 1, configural invariance; Model 2, metric invariance; Model 3, scalar invariance; Model 4, residual invariance. χ^2^, chi-squared test; *df*, degrees of freedom; CFI, comparative fit index; TLI, Tucker-Lewis index; SRMR, standardized root mean square residual; RMSEA, root mean square error of approximation.

Mann–Whitney test for scores of BPIm-S between sex, age and educational status level is shown in Table 6. The results showed that there was no significant difference between male and female in the total score (*p* > 0.05), functional limitation (*p* > 0.05) and psychosocial distress (*p* > 0.05) of the scale; People over the age of 30 had a significantly higher total score (*p* < 0.001), functional limitation (*p* < 0.05), and psychosocial distress (*p* < 0.001) on the scale than those under the age of 30 did; likewise, those with a medium or low education level had a significantly higher total score (*p* < 0.001), functional limitation (*p* < 0.001), and psychosocial distress (*p* < 0.001) than those with a high education level did.

**TABLE 6:** Scores of the BPIm-S between genders, age and different education status groups.

The BPIm-S	Male	Female	Z-scores^a^	>30	≤30	Z-scores^a^	High education	Medium and low education	Z-scores^a^
Total	22 (13, 30)	25 (14, 31)	−1.46	26 (15, 35)	18 (13, 26)	-5.14***	17 (13, 26)	28 (18, 37)	−7.07***
Functional limitation	7 (5, 10)	9 (5, 11)	−1.38	10 (5, 12)	7 (5, 10)	-2.49*	7 (5, 10)	10 (5, 12)	−3.83***
Psychosocial distress	15 (8, 20)	16 (8, 20)	−1.39	16 (9, 24)	11 (8, 16)	-5.82***	10 (8, 16)	18 (12, 24)	−7.80***

^a^Comparison between scores by Mann–Whitney test.

*p < 0.05, ***p < 0.001.

### 3.8. Concurrent validity

Concurrent validity was evaluated through comparisons of the final 13-item version of the BPIm-S questionnaire scores with BPI-F, MOPDS, GAD-7, PHQ-9 (Table 7). Correlations of BPIm-S scores with the BPI-F, MOPDS, GAD-7, PHQ-9 ranged from 0.554 to 0.781 (*p* < 0.001). The BPIm-S exhibited adequate-to-good concurrent validity in relation to this scale.

**TABLE 7:** Correlations of the BPIm-S score with scales for chronic pain interference, pain severity, and other psychosocial variables.

The BPIm-S	BPI-F	MOPD	PHQ-9	GAD-7
Functional limitation	0.628***	0.622***	0.598***	0.554***
Psychosocial distress	0.687***	0.744***	0.781***	0.743***
Total	0.710***	0.749***	0.772***	0.729***

BPI-F, the brief pain inventory-facial; MOPDS, Chinese version of Manchester orofacial pain disability scale; GAD-7, the anxiety disorder assessment; PHQ-9, the patient health questionnaire-9.

***p < 0.001.

### 3.9. Clinical responsiveness

Responsiveness was evaluated in 12 OFP patients who underwent the hot-pressed treatment. After receiving therapy, patients demonstrated considerable improvements on the BPIm-S (total scores, functional limitation scores, and psychosocial distress scores), BPIm-F, MOPD, PHQ-9, and GAD-7 (Table 8).

**TABLE 8:** Responsiveness evaluated in the 12 patients of the hot pressed treatment group.

	Median (interquartile)		
**Scale**	**Before treatment**	**After hot pressed treatment**	**Z-scores^a^**
BPIm-S (total scores)	18.00 (14.00, 25.75)	4.00 (2.00, 5.00)	−3.062**
BPIm-S (functional limitation scores)	7.50 (5.00, 10.00)	2.50 (2.00, 3.00)	−3.063**
BPIm-S (psychosocial distress scores)	10.50 (9.00, 15.50)	1.00 (0.00, 2.00)	−3.063**
BPI-F	28.00 (21.00, 29.75)	8.50 (1.00, 9.75)	−3.064**
MOPDS	29.50 (27.00, 37.50)	13.50 (2.75, 19.00)	−3.061**
PHQ-9	13.00 (9.50, 17.00)	2.50 (1.25, 4.00)	−3.071**
GAD-7	8.00 (7.25, 13.00)	2.50 (0.50, 3.75)	−2.983**

BPI-F, the brief pain inventory-facial; MOPDS, Chinese version of Manchester orofacial pain disability scale; GAD-7, the anxiety disorder assessment; PHQ-9, the patient health questionnaire-9.

^a^Comparison between scores by Wilcoxon signed-rank test.

**p < 0.01.

## 4. Discussion

Patients with OFP have reported worse health-related quality of life due to the condition’s detrimental impact on their ability to do daily activities and their emotional wellbeing (Aguiar et al., 2021). The absence of a Chinese scale able to assess the impact of OFP patients further confounded difficult for doctors to make individualized clinical treatment. Exploring an accurate instrument to assess the physical and psychosocial impairment of OFP was critical. It was the first time to develop the BPIm-S to assess the OFP health life related functional-psychosocial quality through the exploratory sequential research design, which also was proved to be an appropriate PROMS instrument for OFP clinical studies.

Based on the biopsychosocial model suggested by ICOP guidelines and extensive review of the literature on the OFP, we developed the Chinese Version of the BPIm-S in patients with OFP through the principle of item selection, with preliminary and further screening of the items by patients, as well as two rounds of evaluation using the Delphi method. Fifty patients with OFP were asked to review the scale for face validity, and the results showed that two questions had “not relevant” responses from more than 70% of the patients. Because doing housework not always was a daily activities (Platt et al., 2020), and increasing the mealtime always was influenced by work or others (Habib et al., 2020). Experts further verified the construct validity through their experience and deleted two items that are not related to OFP in the sociopsychological dimension. Finally, 13 items across 2 components (functional limitation and psychosocial distress) of the evaluation index system were selected to create the BPIm-S.

Since the BPIm-S was firstly developed and used in the Chinese clinical population, we firstly explored its reliability to evaluate the internal consistency (Rattray and Jones, 2007). The inter-total correlations between the BPIm-S items demonstrated their consistency, usefulness, and lack of redundancy. According to the results of Cronbach’s α, the scale’s internal consistency was quite high (Bonett and Wright, 2015). Our results showed that the BPIm-S with 13 items was still a reliable and stable instrument for measuring and assessing the influence of Chinese patients with OFP, which guaranteed follow-up psychometric research.

We further explored the factor structure of the BPIm-S, and the EFA analyses identified two domains, functional limitation and psychosocial distress. Factor 1 is labeled “functional limitation” because it contains five items that reflect the functional limitation caused by symptom onset in OFP patients. Examples of such behaviors include mouth opening restrictions, painful eating. This factor corresponds to previous studies describing physical disabilities associated with OFP patients. Factor 2 contains eight items and is labeled as “psychosocial distress” because it involves distress that mainly embodies the psychosocial dimension of OFP patients. Also, we also proved the internal convergence validity through CR and AVE, suggesting that the two-factor structure in the BPIm-S did not intersect and could be calculated as independent dimensions. We further used CFA to evaluate the two-factor structure instrument of the FPIm-S, The CFA verified the EFA output and provided an initial proof of the construct validity of the FPIm-S.

The novel contribution of the present study lies in the analysis of measurement invariance, which previously had been lacking. Measurement invariance was also established across gender, age, and educational status groups to further support the reliability of the 2-factor model. The BPIm-S exhibited high levels of configural, metric, scalar, and residual invariance across male and female OFP patient samples, as validated by our MGCFA analysis. Two measures evaluating functional limitation and psychosocial distress showed that BPIm-S was conceived identically in women and men, lending credence to the notion of configural invariance. Metric invariance was also supported, which meant that the same units of measurement apply to both sexes. Further, the current scalar invariance setup suggested that disparities in scores between males and females may be understood as representing real group differences in latent variables, which offered a common baseline for both sexes. It is only when the units and reference points are the same that comparisons across groups become relevant. Consequently, the assumption of metric equivalence and scalar equivalence in order to do a latent mean comparison (Hermida, 2015). Finally, the cross-gender difference in latent variable variation was mirrored in the support for the residual invariance across both women and men (Shah and Goldstein, 2006). The outcomes depend on age and education level in a similar fashion. In conclusion, this study’s findings corroborate the measurement invariance of the FPBIm-S, suggesting its efficacy and interpretability across demographics such as gender, age, and education level. The findings of the present study allow researchers to apply the HRFS in a wider variety of research designs. Measurement invariance is an important prerequisite for comparisons between groups.

We utilized the Mann–Whitney to compare means across categories such as gender, age, and level of education, there was no significant difference in the results of the measurement invariance test between the gender. Males and females did not vary significantly in terms of total score, functional limitation component, or psychological distress dimension, according to these findings. However, previous studies have shown that the prevalence and symptoms of OFP tend to be higher among females than among males (Cairns, 2007). This is inconsistent with the findings of this study. Since the measurement invariance between the gender has already been studied. In this case, since we can rule out potential interference caused by gender differences, the results can be relied upon. In terms of age, the total score, functional limitation, and psychosocial distress of the scale of people over 30 years old were significantly higher than those of people under 30 years old. This is consistent with previous studies (Salman Roghani et al., 2019). In terms of educational status, the total score, functional limitation and psychosocial distress of the medium education and low education population were significantly higher than those of the High education population. A similar result has been obtained in a previous study, this may be because people with higher educational status have higher self-perception ability in OFP management (Taqi et al., 2021).

To test the concurrent validity, spearman correlation coefficients of BPIm-S and BPI-F, MOPD, PHQ-9, and GAD-7 scores were calculated. All scales correlate well with the BPIm-S, indicating adequate concurrent validity. This study further investigated the responsiveness of the BPIm-S and BPI-F, MOPD, PHQ-9, and GAD-7 scores to clinical outcomes in OFP patients after hot compress treatment. The BPIm-S was shown to be sensitive to changes in clinical outcomes, indicating that it is a valid tool for gauging improvement in the health of OFP patients and the efficacy of treatment. The results of this investigation give further support for the use of the FPIm-S, expanding the findings of earlier studies in both applied and research contexts, and so contribute to the ongoing validation of the FPIm-S in clinical settings.

Assessment of clinical outcomes with PROMs is increasingly important in the evaluation of patients (Dawson et al., 2010). The process of the BPIm-S completion prompts patients to reflect on their health and in doing so, patients develop a deeper understanding of how their condition affects them. By answering the questions on the BPIm-S, patients are prompted to think about their health and get insight into the impact of their disease. Proactive use of the BPIm-S during follow-up may enhance patient involvement leading to increased satisfaction with care. Work still needs to be done to understand how the BPIm-S can be utilized effectively to improve patient outcomes.

Despite the thorough approach used to create a psychometrically sound scale, a few research limitations were discovered that must be carefully considered. A small sample size was the limitation. More measurement invariance of clinical samples can be verified, such as work status, etc. After that, a longitudinal study can be conducted with the target population to study the reliability of the FPIm-S for OFP disease follow-up.

## 5. Conclusion

This research examines physical and psychosocial search using a standard constructed and tested measure, a review of OFP, concept analysis, and structured interviews. The tool consists of 13 questions divided into two categories: Functional limitation and psychosocial distress. The instrument, BPIm-S, displayed strong psychometric qualities and clinical responsiveness; hence, it may be used to investigate and quantify the distress in OFP patients’ functional limitation and psychological distress.

## Data availability statement

The raw data supporting the conclusions of this article will be made available by the authors, without undue reservation.

## Ethics statement

The studies involving human participants were reviewed and approved by the Experimentation and Ethics Committee of the Second Xiangya Hospital of Central South University. The patients/participants provided their written informed consent to participate in this study.

## Author contributions

YF and Z-YO-Y: conceptualization, methodology, resources, and writing—original draft preparation. D-DX and Y-FY: validation. Z-YO-Y, YF, and N-XC: formal analysis. Z-YO-Y and X-LS: investigation. D-DX, YC, and JZ: data curation. YF, Y-QZ, and Z-YO-Y: writing—review and editing. YF, BF-K, and JZ: visualization. YG and Y-ZF: supervision, project administration, and funding acquisition. All authors read and agreed to the published version of the manuscript.
