# Supplementary material for: Development, validation and psychometric evaluation of the Chinese version of the biopsychosocial impact scale in orofacial pain patients
Source: Front Psychol. 2023 Mar 7;14:1101383. doi: 10.3389/fpsyg.2023.1101383 (PMC10029920; doi:10.3389/fpsyg.2023.1101383)
Supplement: Supplementary file 1 [file Table_1.docx]

Supplementary Material

Development, validation and psychometric evaluation of the Chinese version of the biopsychosocial impact scale in orofacial pain patients Ze-Yue Ouyang ^1^ †, Yao Feng^1^ †, Dong-Dong Xie ^2^, Yi-Fan Yang ^1^, Yun Chen ^1^, Ning-Xin Chen ^1^, Xiao-Lin Su ^1^, Jie Zhao ^1^, Ya-Qiong Zhao ^1^, Yun-Zhi Feng ^1^, Yue Guo ^1*^

†These authors have contributed equally to this work and share first authorship

*** Correspondence:**

Yun-zhi Feng：fengyunzhi001@csu.edu.cn

Yue Guo: guoyue@csu.edu.cn

**Table S1. Search strategy.**

| **Search strategy**  **PubMed** |
| --- |
| ("orofacial pain"[Title]) AND ("assessment"[All Fields] OR "measures"[All Fields] OR "assess"[All Fields] OR "measure"[All Fields] OR "evaluation"[All Fields] OR "evaluate"[All Fields])  Filters applied: Free full text, Clinical Study, Clinical Trial, Guideline, Meta-Analysis, Observational Study, Randomized Controlled Trial, Review, Systematic Review. |
| **China National Knowledge Infrastructure (CNKI)** |
| SU='orofacial' AND SU='pain' AND FT= ('measures' + 'measure' + 'assess' + 'assessment' + 'evaluate' + 'evaluation')  Filters applied: Chinese/English Extended |
| **Wanfang database**  （Subject:(orofacial pain)) and Date:1900-* |
| **Web of Science** |
| ("orofacial pain") AND ("assessment" OR "measures" OR "assess" OR "measure" OR "evaluate" OR "evaluation") |

**Table S2. Final version of the Biopsychosocial Impact Scale (BPIm-S).**

在过去三个月中，你的脸部、口腔或下巴是否有疼痛？

有 没有

| 因我的脸部、口腔或下巴疼痛 | 以下情况在过去三个月中是否适用于我，以及它对我的生活的影响程度 (请根据你的实际情况在线上打勾) | | | |
| --- | --- | --- | --- | --- |
|  | 无影响 | 轻度影响 | 中度影响 | 严重影响 |
| 1. 我很难长时间说话： | - | - | - | - |
| 2. 我不能张大嘴巴： | - | - | - | - |
| 3. 我不能碰脸 | - | - | - | - |
| 4. 我不能吃硬的食物： | - | - | - | - |
| 5. 我不能微笑或大笑： | - | - | - | - |
| 6.我的情绪状况受影响： | - | - | - | - |
| 7. 我入睡受影响： | - | - | - | - |
| 8. 我很难集中精神 ： | - | - | - | - |
| 9. 我不再享受食物： | - | - | - | - |
| 10. 我对任何事情都不能产生热情： | - | - | - | - |
| 11. 我感觉疲惫无力： | - | - | - | - |
| 12. 我感觉自己得了严重的疾病： | - | - | - | - |
| 13. 我宁愿自己待着： | - | - | - | - |

Have you had any pain in your face, mouth or jaws in the past three month?

Yes No

Here are some statements about problems that people have because of pain in their face, mouth or jaw

For each statement, please indicate whether this applies to you in the last three months and the extent to which it has affected your life.

| Because of pain in my face, jaws or mouth: | During the past three month this has applied to me: (please tick on line under appropriate statement) | | | |
| --- | --- | --- | --- | --- |
|  | Does not affect at all | Mildly affect | Moderately affect | Severely affect |
| 1.I find it hard to talk for long periods of time | - | - | - | - |
| 2.I cannot open my mouth as wide as I could | - | - | - | - |
| 3.I cannot touch my face | - | - | - | - |
| 4.I cannot eat hard foods | - | - | - | - |
| 5.I find it difficult to smile or laugh | - | - | - | - |
| 6.I feel emotionally affected by the situation | - | - | - | - |
| 7.I have difficulty falling asleep | - | - | - | - |
| 8.I have found it difficult to concentrate | - | - | - | - |
| 9.I no longer enjoy my food | - | - | - | - |
| 10.I can't get enthusiastic about anything | - | - | - | - |
| 11.I feel tired | - | - | - | - |
| 12.I am worried that I may have a serious illness | - | - | - | - |
| 13.I would rather be by myself | - | - | - | - |

# Table S3. OFP patient distribution by gender, age, education.

|  | Age (years) | | Gender | | Education status | |
| --- | --- | --- | --- | --- | --- | --- |
|  | > 30 | ≤ 30 | Male | Female | High education  (Undergraduate and above) | Medium and low education  (High school and Junior high school) |
| Migraine | 80 | 79 | 62 | 97 | 84 | 75 |
| Trigeminal neuralgia | 11 | 5 | 6 | 10 | 8 | 8 |
| Tension and maxillofacial headache | 41 | 23 | 27 | 37 | 26 | 38 |
| Cluster headache | 79 | 9 | 30 | 58 | 16 | 72 |
| Temporomandibular joint disorder | 7 | 38 | 21 | 24 | 40 | 5 |
| Burning mouth syndrome | 3 | 3 | 2 | 4 | 4 | 2 |
| Toothache | 61 | 70 | 59 | 72 | 92 | 39 |
